# *In vitro* Cortical Network Firing is Homeostatically Regulated: A Model for Sleep Regulation

**DOI:** 10.1038/s41598-018-24339-6

**Published:** 2018-04-19

**Authors:** Sohrab Saberi-Moghadam, Alessandro Simi, Hesam Setareh, Cyril Mikhail, Mehdi Tafti

**Affiliations:** 1 Center for Integrative Genomics, Faculty of Biology and Medicine, University of Lausanne, Génopode, 1015 Lausanne, Switzerland; 2 Laboratory of Computational Neuroscience, School of Computer and Communication Sciences, EPFL, 1015 Lausanne, Switzerland; 3 Department of Physiology, Faculty of Biology and Medicine, University of Lausanne, Bugnon 7, 1005 Lausanne, Switzerland

## Abstract

Prolonged wakefulness leads to a homeostatic response manifested in increased amplitude and number of electroencephalogram (EEG) slow waves during recovery sleep. Cortical networks show a slow oscillation when the excitatory inputs are reduced (during slow wave sleep, anesthesia), or absent (*in vitro* preparations). It was recently shown that a homeostatic response to electrical stimulation can be induced in cortical cultures. Here we used cortical cultures grown on microelectrode arrays and stimulated them with a cocktail of waking neuromodulators. We found that recovery from stimulation resulted in a dose-dependent homeostatic response. Specifically, the inter-burst intervals decreased, the burst duration increased, the network showed higher cross-correlation and strong phasic synchronized burst activity. Spectral power below <1.75 Hz significantly increased and the increase was related to steeper slopes of bursts. Computer simulation suggested that a small number of clustered neurons could potently drive the behavior of the network both at baseline and during recovery. Thus, this *in vitro* model appears valuable for dissecting network mechanisms of sleep homeostasis.

## Introduction

Sleep regulation is one of the most intriguing topics in the field of neuroscience. Sleep is a complex brain state and is believed to be necessary for normal functioning during waking. Two main stages constitute sleep: rapid eye movement sleep (REM or paradoxical sleep), and non-rapid eye movement sleep (NREM or slow wave sleep, SWS). NREM sleep is characterized by high amplitude and low frequency quasi-synchronous cortical network activity^1^. The NREM network oscillations are divided in the electroencephalogram (EEG) slow oscillation (<1 Hz)^2,3^ and slow wave or delta activity (EEG power density between 0.5–4 Hz, SWA)^1,4^. Sleep is homeostatically regulated. Prolonged periods of spontaneous wakefulness or sleep deprivation lead to an increased sleep need that is manifested in a proportional increase in EEG SWA and an increased incidence of high amplitude slow oscillations during recovery sleep^5–7^. Homeostatic regulation of sleep is not limited to an increase in EEG SWA in mammalian species but extends to an increased sleep duration and reduced response to external stimuli in nearly all species so far studied^8–10^. Nevertheless, the underlying cellular, network, and molecular mechanisms of sleep homeostasis are poorly understood.

Intracellular recordings of cortical neurons during SWS or anesthesia revealed a robust slow oscillation characterized by a period of active firing (UP state) followed by a long-lasting period of neuronal silence (DOWN state)^2,11^. This pattern of network activity can be reliably recorded during SWS in intact animals (by local field potential “LFP” and multiunit activity recordings) and in humans (by the EEG)^3,12–17^. Interestingly, this slow oscillation occurs spontaneously in thalamic inactivated cortical regions or isolated cortical slabs^18^, cortical slices^19^, or even in mature cortical cultures^20–24^. By using multielectrode arrays (MEA) and mouse primary cortical cultures, we showed that not only these dish-wide slow oscillations can be recorded for long periods of time but that cultures can be stimulated by a waking chemical cocktail (hereinafter called “CCK”, including monoaminergic, glutamatergic, cholinergic, and orexinergic neurotransmitters or agonists) to induce tonic firing that returns to the default synchronous burst firing 24 h later^22^. Notably, stimulated cultures show remarkably similar transcriptional and metabolic changes as cortical tissues of animals subjected to 6 h of sleep deprivation^22^. One important finding in this study, which was confirmed and extended by Kaufman *et al*.^25^, is that even continuous stimulation of such cultures cannot prevent the invariable return of slow oscillations, strongly indicating that homeostatic processes are activated to compensate for imposed tonic firing. Here we performed continuous recording of mouse cortical cultures before stimulation by two different concentrations of our CCK and 24 hours later during recovery. Detailed analysis of burst firing in these preparations revealed a dose-depended homeostatic response of the network activity, which showed remarkable similarities to homeostatic regulation of cortical activity during physiological sleep *in vivo*.

## Results

**Figure 1:**
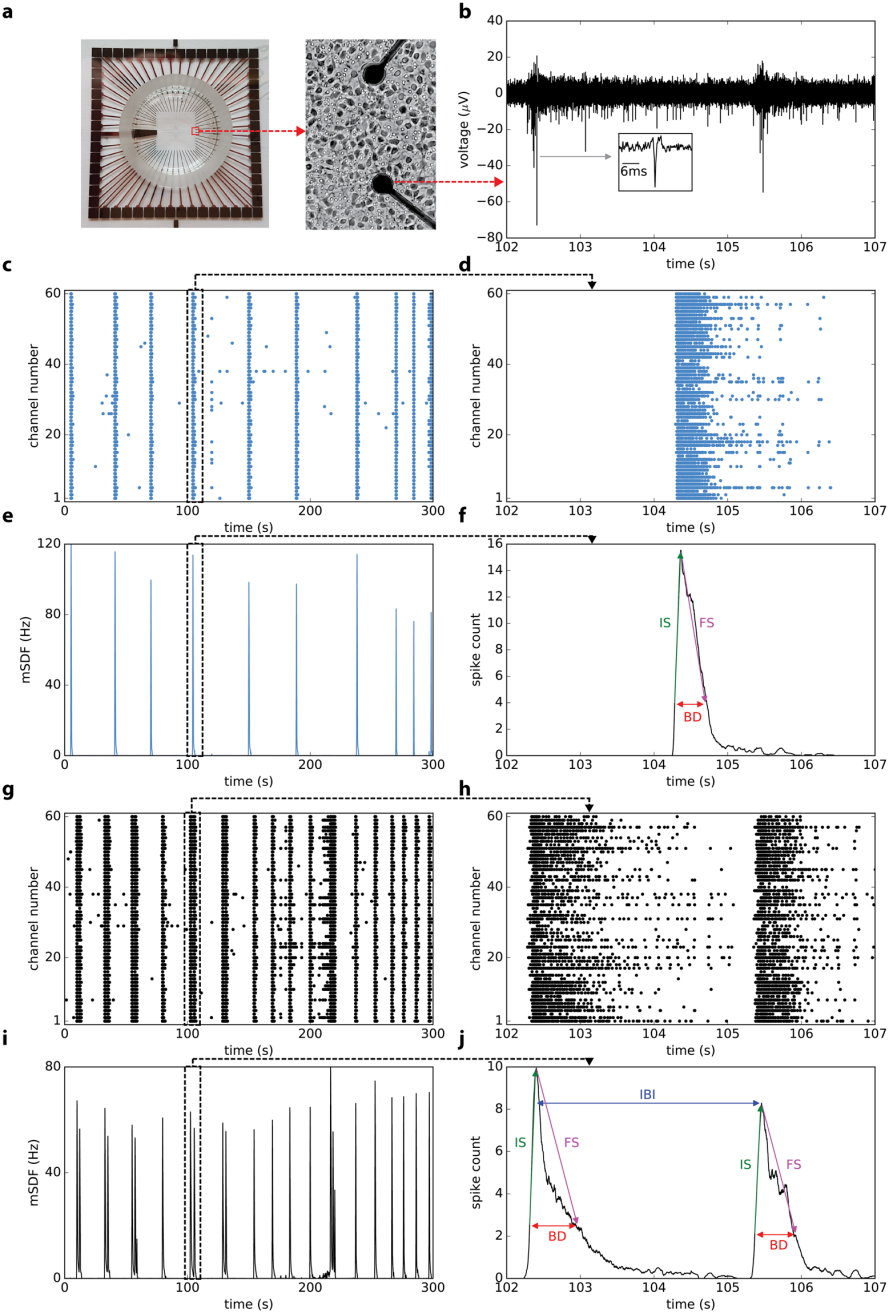
Synchronized burst firing and burst characteristics in representative MEA recordings of a 14DIV mouse cortical culture at baseline and during recovery. (**a**) Picture of an MEA with 60 electrodes and zoomed figure of two electrodes with neuronal culture. (**b**) Five seconds of raw MEA recording. The inset shows a typical spike at higher resolution. Five minutes raster plots and mean spike density function (mSDF) recorded in one culture at baseline (**c**,**e**) and during recovery (**g**,**i**). Zoomed figures of raster plots (**d**,**h**) and mean spike density (**f**,**j**) provide higher resolutions. Automatic detection of bursts with their parameters are shown in (**f**,**j**). IS: initial slope, FS: final slope, BD: burst duration, and IBI: inter-burst interval.

**Figure 2:**
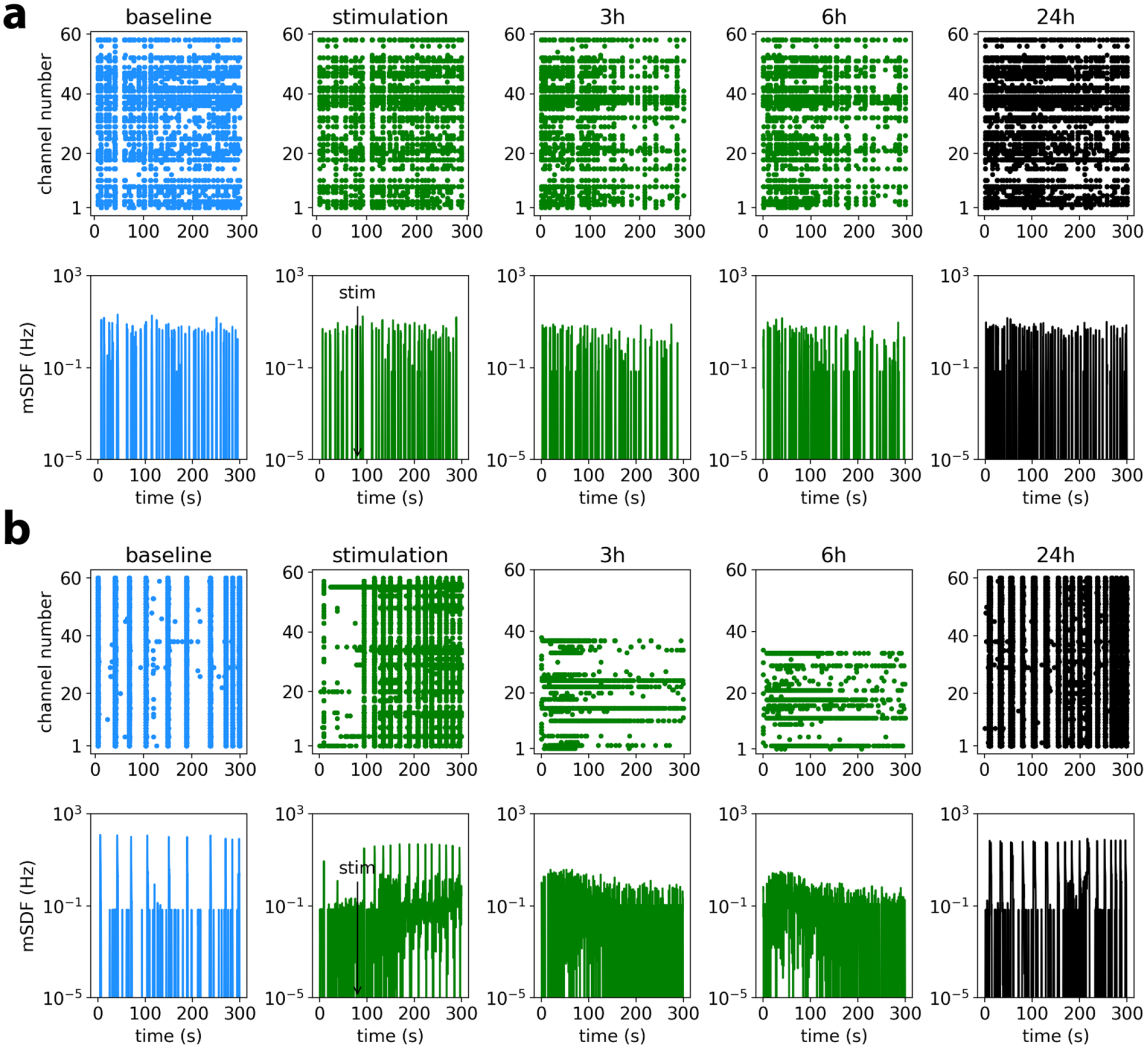
Time course of the network firing behavior. A representative MEA recording at baseline and following stimulation with sham (**a**) or a cocktail of neuromodulators (1CCK, **b**). Tope panels show the raster plots and lower panels the mean spike density functions. Stimulation results in the disappearance of bursting activity which is replaced by tonic firing. The bursting activity recovers after 24 h. The lower panels in b are presented in log scale to visualize the low amplitude high frequency tonic activities after stimulation.

Developing cortical networks *in vitro* show initially a random firing that gradually is transformed into a synchronized bursting pattern within a two weeks period (14 days *in vitro* “DIV”) and remains stable thereafter^26,27^. Synchronized bursting *in vitro* tightly correlates with the membrane depolarization (UP state) of single neurons by intracellular recordings^25^ and show similarities to the firing activity of cortical neurons during SWS sleep *in vivo*^11^. Also, similar to the *in vivo* activity, the burst-pause firing *in vitro* occurs at low frequency (typically between 0.1 to 0.5 Hz)^22^. Examples of 5-minute recordings of a culture at 14DIV before and 24 h after stimulation with our waking neuromodulator cocktail^22,28^, are shown in Fig. 1. Note that, the waking cocktail was added to each culture but the medium was not changed (no washing) during the recordings. Bursting activity was characterized as described in Methods and the burst parameters analyzed here are indicated in Fig. 1f,j. CCK (Fig. 2b) but not sham stimulation (Fig. 2a) rapidly suppressed burst firing. We hypothesized that similar to early phase of sleep, signs of recovery must be seen at the re-emergence of burst activity (24 h after stimulation).

### Spectral analysis, burst duration and interburst interval

**Figure 3:**
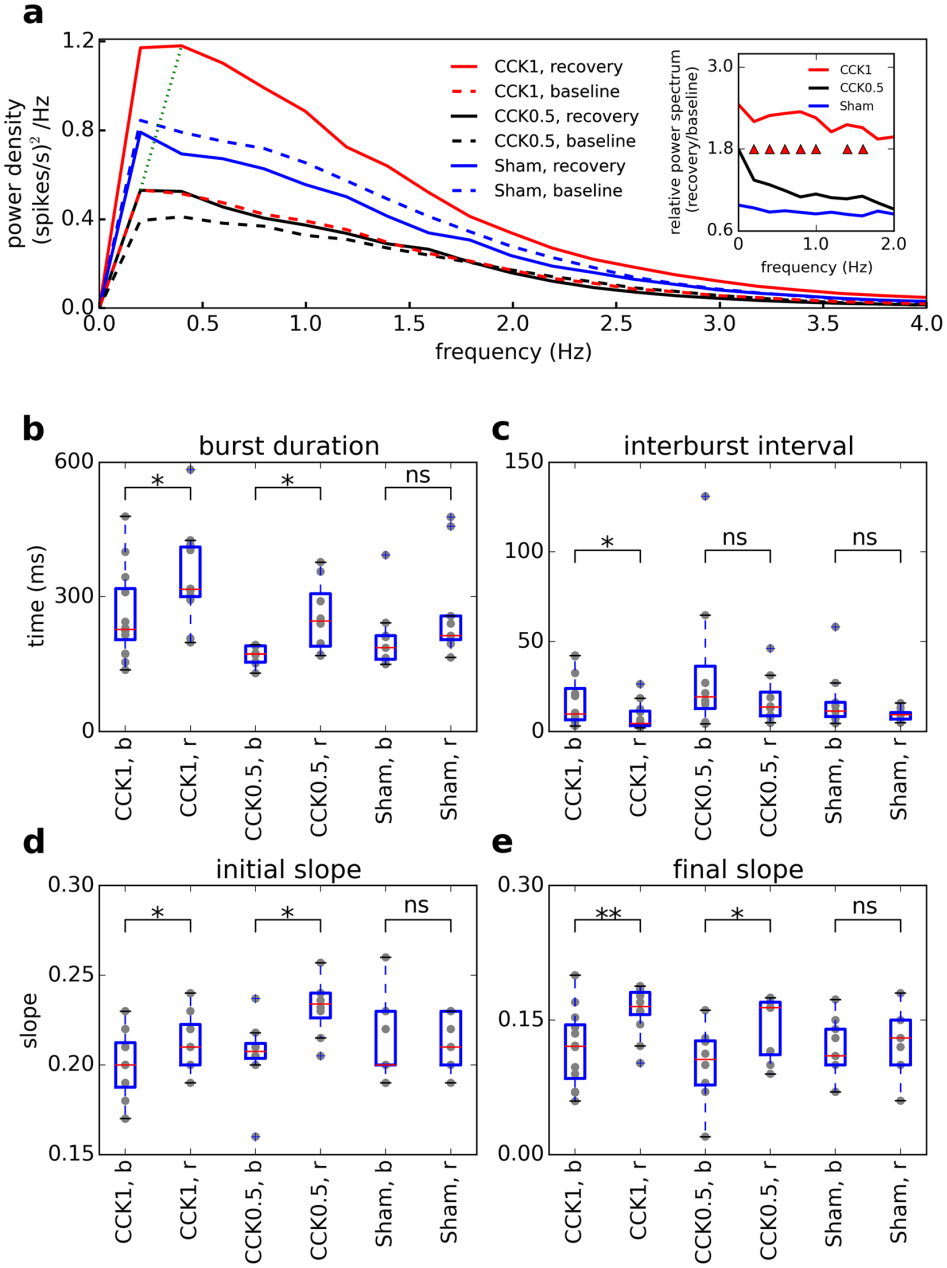
Spectral and burst properties of *in vitro* cortical networks at baseline and during recovery after neuromodulatory or sham stimulation. (**a**) Spectral power density of bursting activity of cultures during baseline (blue lines) and recovery (black lines) for 1CCK (n = 12), 0.5CCK (n = 8), and sham stimulation (n = 9). The power spectrum shows an increase with a shift toward higher prominent peak during recovery compared to baseline (the green dotted line connects the maximum power at baseline to recovery for 1CCK). The inset indicates the relative changes in power densities during recovery (triangles indicate a significant increase for 1CCK p < 0.05; post hoc Tukey test after 2-way ANOVA with repeated measures). (**b**) Burst Duration (BD) is significantly longer and (**c**) the inter-burst interval (IBI) is shorter during recovery for 1CCK and 0.5 CCK while there is no change in sham stimulated cultures. (**d**) Initial and **e** final slopes are significantly increased after 1CCK and 0.5 CCK stimulation while no change is observed after sham stimulation. Each dot represents a single culture. **Bb**: baseline, r: recovery, ns: non-significant, *p < 0.05, **p < 0.01; paired t-test on relative values (to the sham condition).

Slow waves during NREM sleep arise from a synchronized occurrence of UP and DOWN states among large cortical neuronal populations^11^. More specifically, the negative segment of the slow waves coincides with network silence^29^. This activity can be approximated by the envelope of bursts (spike density function) as shown in Fig. 1e,i. Time series of smoothed firing activities were subjected to a fast Fourier transform (FFT) analysis. Cultures recorded 24 h after stimulation showed a dose-dependent increase in spectral power below 1.75 Hz (Fig. 3a). In addition, a right shift in the dominant frequency was observed (Fig. 3a). The increase in power density can result from an increase in the incidence of bursts and/or an increase in their amplitude.

We therefore calculated both the duration and the inter-burst interval as outlined in Fig. 1j. None of the burst parameters at baseline differed significantly between sham, 1 and 0.5 CCK (one-way ANOVA, p > 0.1). We therefore normalized these parameters by dividing to the mean of the sham condition (both at baseline and recovery) followed by paired t-test to detect the effect of stimulation Fig. 3a–d). It was proposed that a higher homeostatic need for recovery results in less neuronal activity^30^. This can be achieved by a decrease in the incidence of UP states and/or longer neuronal silent periods (DOWN states). *In vivo* intracellular recordings of cortical neurons during recovery sleep after sleep deprivation are lacking, but our results clearly show that the burst duration during recovery is significantly increased after both 1 (p < 0.04) and 0.5 (p < 0.02) CCK stimulation and inter-burst interval is significantly decreased after 1CCK stimulation (p < 0.02) (Fig. 3b,c). These findings suggest that higher homeostatic pressure *in vitro* results in an increased incidence of bursts (UP state) and a decreased inter-burst interval (DOWN state).

### Burst slopes

*In vivo* EEG recordings in humans and LFP recordings in rats, as well as computational simulations, indicated that the right shift in the major slow frequency power density is related to steeper slopes of slow waves^16,17^. Changes in the slope of slow waves are caused by the synchronization of neuronal activity in the network, so that faster synchronization events lead to steeper slopes^31^. In our recordings the shape of the bursts depends on the firing activity recorded across electrodes (spike density) and therefore the height of the burst envelops is limited by the density of spikes recorded and the number of active electrodes. We therefore calculated the slopes of the rising and decaying segments of the burst envelops (Fig. 1j) and compared them between baseline and recovery (24 h after stimulation) recordings. Both initial and final slopes significantly increased during recovery and these changes were larger after 1CCK (initial slope, p < 0.05, final slope, p < 0.01) than after 0.5CCK stimulation (initial slope, p < 0.05, final slope, p < 0.05), while no changes were observed after sham stimulation (initial slope, p = 1, final slope, p > 0.40) (Fig. 3d,e). Note that the final slope, which we found highly significantly steeper, is equivalent to the first segment of negative waves as recorded by the LFP and the EEG.

### Cross correlation

**Figure 4:**
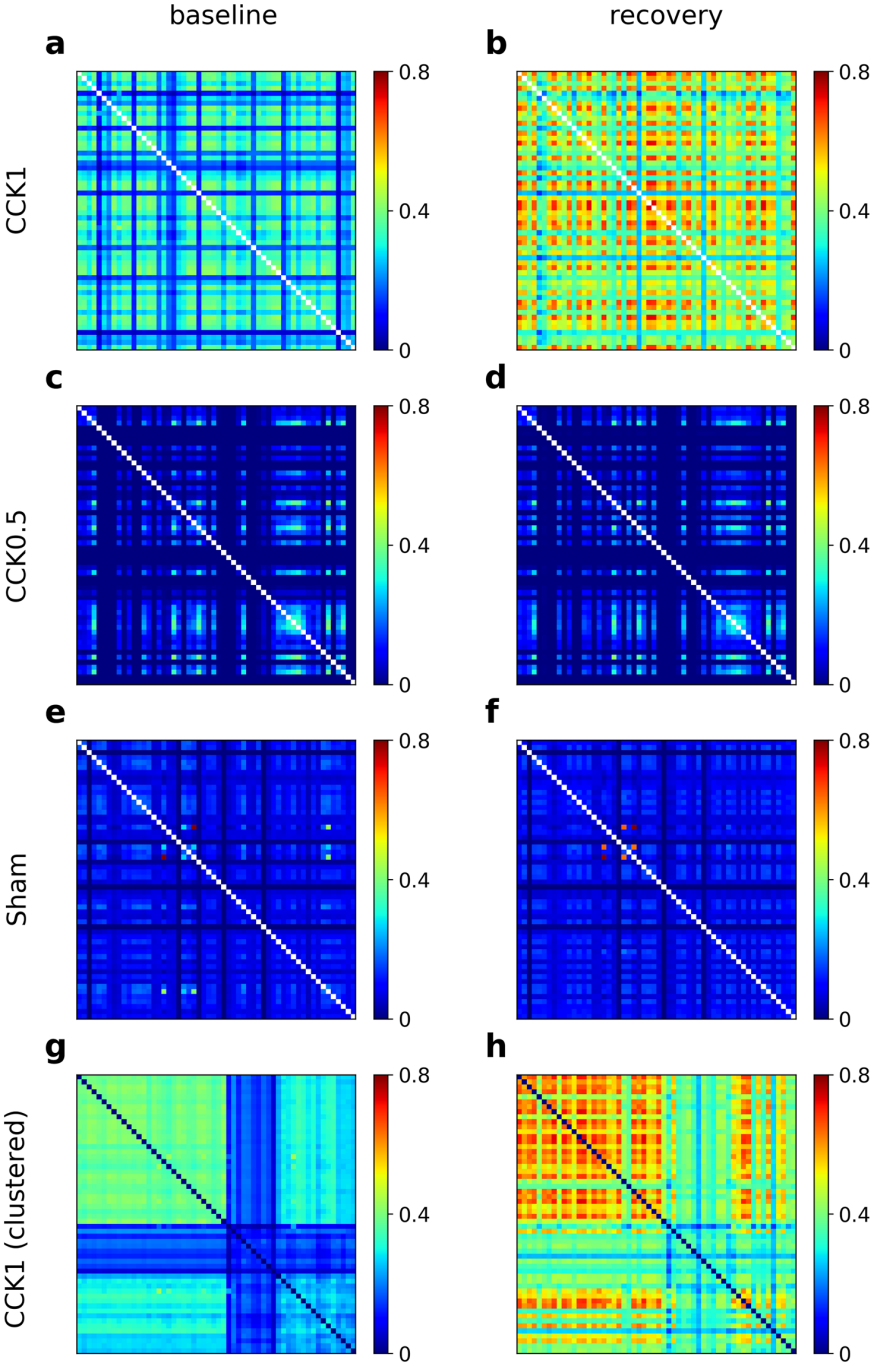
Cross-correlations between paired MEA channels. (**a**–**f**) Representative cross-correlation matrices of spike trains between paired MEA channels at baseline (**a**,**c**,**e**) and during recovery (**b**,**d**,**f**) after 1CCK (**b**), 0.5CCK (**d**), and sham (**f**) stimulation. The population channel activity reveals higher temporal cross-correlation between discharge times of spikes during recovery for 1CCK (**b**) where the level of synchrony is increased. There is no change between baseline and recovery after 0.5CCK (**d**) and sham stimulation (**f**). Hierarchical clustering of the same cross-correlation matrices in (**a**,**b**) are shown in (**g**,**h**). Note the presence of clusters with different cross-correlations at baseline and during recovery.

The homeostatic response after prolonged wakefulness *in vivo*, is manifested during recovery sleep in an increase in neuronal synchronization across large cortical regions. In our preparations, the level of synchronization in spatiotemporal neural network can be reliably measured by cross-correlation between electrode pairs. As shown in Fig. 4, cultures recorded 24 h after stimulation at 1CCK show a higher cross-correlation than baseline, indicating that recovery from stimulation leads to a stronger and larger synchronization across the network. However, the increase in synchronization does not occur across the entire recording dish but is restricted to clusters of electrodes (Fig. 4b). Accordingly, a hierarchical clustering analysis of the cross-correlation matrices clearly shows clusters of paired electrodes with high cross-correlations (Fig. 4g,h).

### Firing rate, burst duration histogram, and neural trajectory

**Figure 5:**
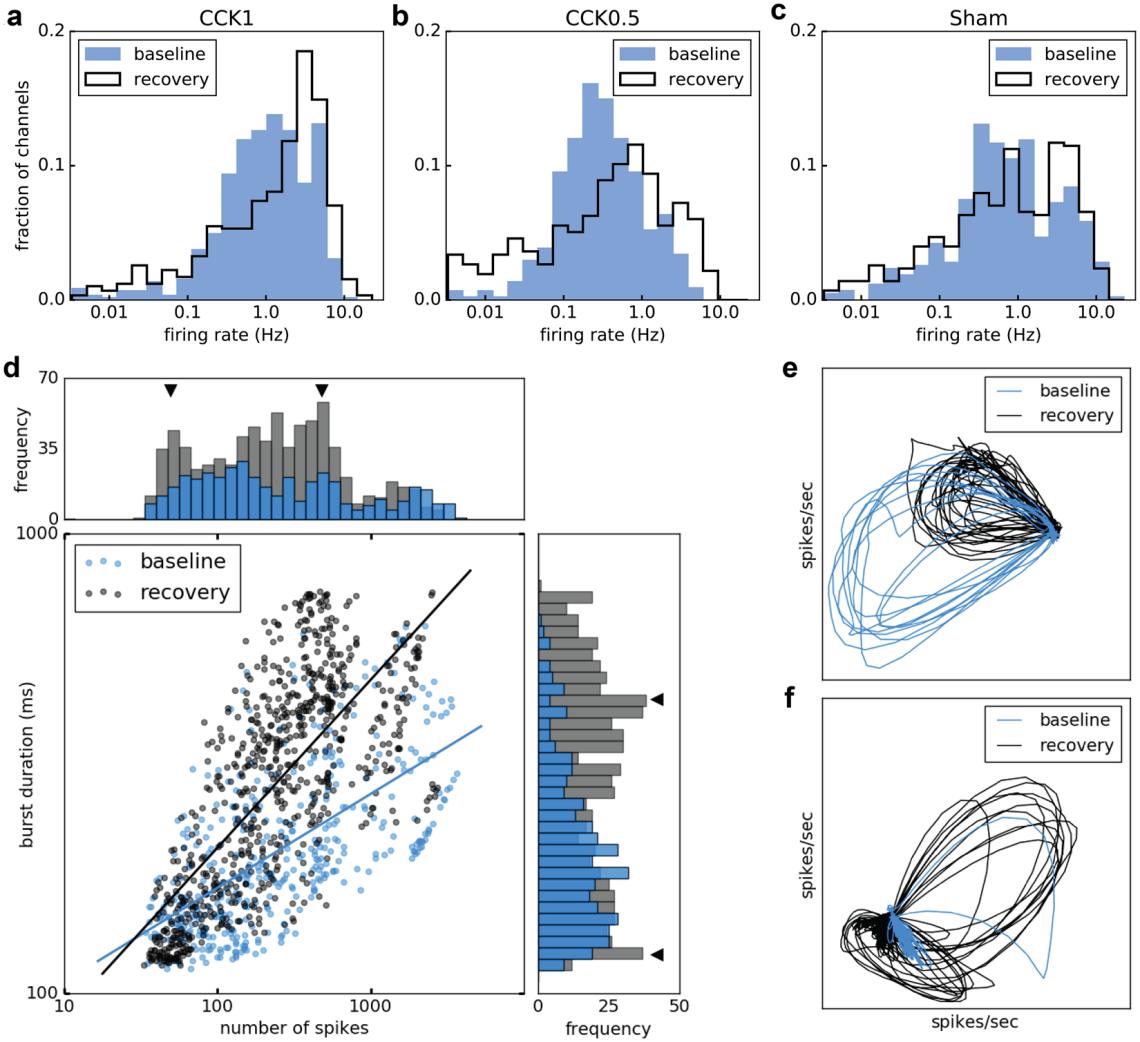
Changes in firing rate during bursts. (**a**–**c**) Firing rate distributions at baseline (blue) and during recovery (black) after stimulation with the neuromodulatory cocktail at 1 and 0.5 CCK or after sham stimulation. More MEA channels present higher spike rate during recovery after 1 and 0.5CCK stimulations. (**d**) Correlation between the burst duration and the number of spikes (log scales) at baseline (blue) and during recovery (black) and the corresponding frequency histograms. Triangles indicate changes in frequency histograms during recovery. (**e**,**f**) Neural trajectories for two representative cultures captured by a 2-d projection of firing rate space. (**e**) The neural trajectory of spontaneous activity in one culture for baseline (blue) and recovery (black). The X and Y axes correspond to projection vectors that are linear combinations of firing rates. Two characteristics are detected: phase and amplitude. The strong phase between up and down states are reflected more in recovery (circle traces) with lower amplitude. (**f**) Increasing number of circle traces with higher amplitude in this culture during recovery (black) compared to lower one in baseline (blue). For both cultures the neural trajectories start from one point locally and propagate in firing rate space with various phases.

Increase in sleep need not only increases synchronization but also increases excitability during the UP state and in susceptible human subjects may lead to seizures^32,33^. Overall, spike rates did not significantly change between the three conditions or between baseline and recovery. Nevertheless, frequency distribution of spike counts indicated more channels with higher spike rates during recovery after 1 and 0.5CCK stimulation (Fig. 5a,b), while no difference was found after sham stimulation (Fig. 5c).

The temporal structure of the network activity was also analyzed by two additional methods. First, there is a positive correlation between burst duration (BD) and the number of spikes across all channels, so that longer bursts recruit more channels with higher spike numbers both at baseline and during recovery (Fig. 5d). Nevertheless, during recovery there is a stronger correlation (between regression lines, p < 0.005, z-score, Fig. 5d) with a clear increase in longer bursts (p < 0.05, cross-tabulation) with higher spike numbers (p = 0.07), resulting in the appearance of a second peak in the distribution of BD and spike numbers (Fig. 5d). Second, to analyze the temporal evolution of the firing rate (network behavior) we used neural trajectory analysis. A neural trajectory describes the time-evolution of network population activity that can be traced over time in the space^34^. By using a 2-d projection of firing rate space, we mapped the network activity, with different phase and amplitude, that starts at a local temporal space (dense central neural activity) and propagates globally over the network. Figure 5e,f show neural trajectories of two representative cultures at baseline and during recovery after 1CCK stimulation. Increased number of black circle traces in both cultures during recovery indicates a strong phasic synchronized activity compared to baseline (blue circle traces). Differences in neural trajectories suggest that not all neurons but selected groups of neurons (local clusters) contribute to the network population activity.

### Simulation of neural network behavior and topology

Sleep is local and use-dependent^35^. Cortical networks or even individual cortical columns that are highly stimulated during wakefulness show higher probability to enter sleep with larger increase in SWA^36–38^. Also, small cortical networks can show signs of sleep in otherwise awake and sleep deprived animals^39^. Therefore, the recruitment of larger cortical areas by small neural clusters might result to the whole network sleeping behavior while individual neurons might not. To test if the network behavior can be predicated by local (cluster) activity shaping the overall network behavior we built several neural networks with different topologies and obtained their behavior at baseline and during recovery using computer simulation. Recovery processes include changes in synaptic weight and network topology (connectivity). Obviously, cortical cultures lack the intact cortical connectivity and each culture is unique in terms of established network. We propose that the structure and topology of neural networks *in vitro* plays an important role in generating the network oscillation and establishing its properties (e.g., duration of bursts or the regularity of oscillations). A network feature which is often used for modeling neural networks is the neural cluster (also called neural assembly). A neural cluster is a subgroup of neurons with dense connectivity or strong synaptic weight. Previous studies showed that embedding neural clusters in a larger network significantly changes the dynamics and behavior of the whole network^40,41^. Here we embedded one or two clusters of excitatory neurons to produce oscillations.

**Figure 6:**
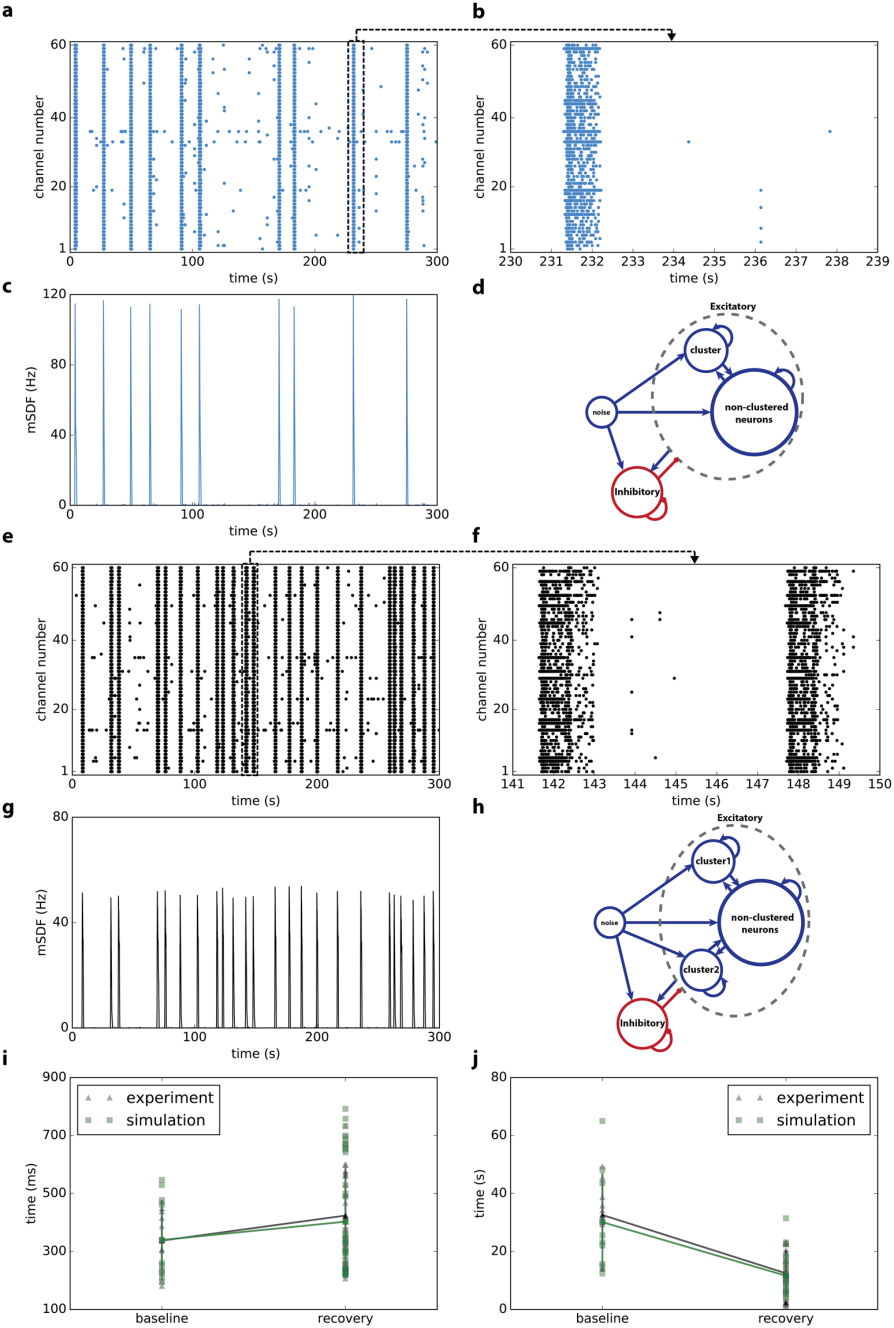
Computer simulation of the network firing behavior of the culture shown in Fig. 1. Network topology changes after the stimulation: while we used only one cluster at baseline (**d**), we assumed that another cluster appeared in recovery (**h**). Simulated raster plots (baseline **a**, recovery **e**) and mean spike densities (baseline **c**, recovery **g**) are similar to experimental data (Fig. 1). A 9 second higher resolution of simulated multiunit activity (baseline **b**, recovery **f**) shows typical bursts. Comparisons of burst durations (**i**) and inter-burst intervals (**j**) between experimental and simulation data indicate a very similar pattern.

**Figure 7:**
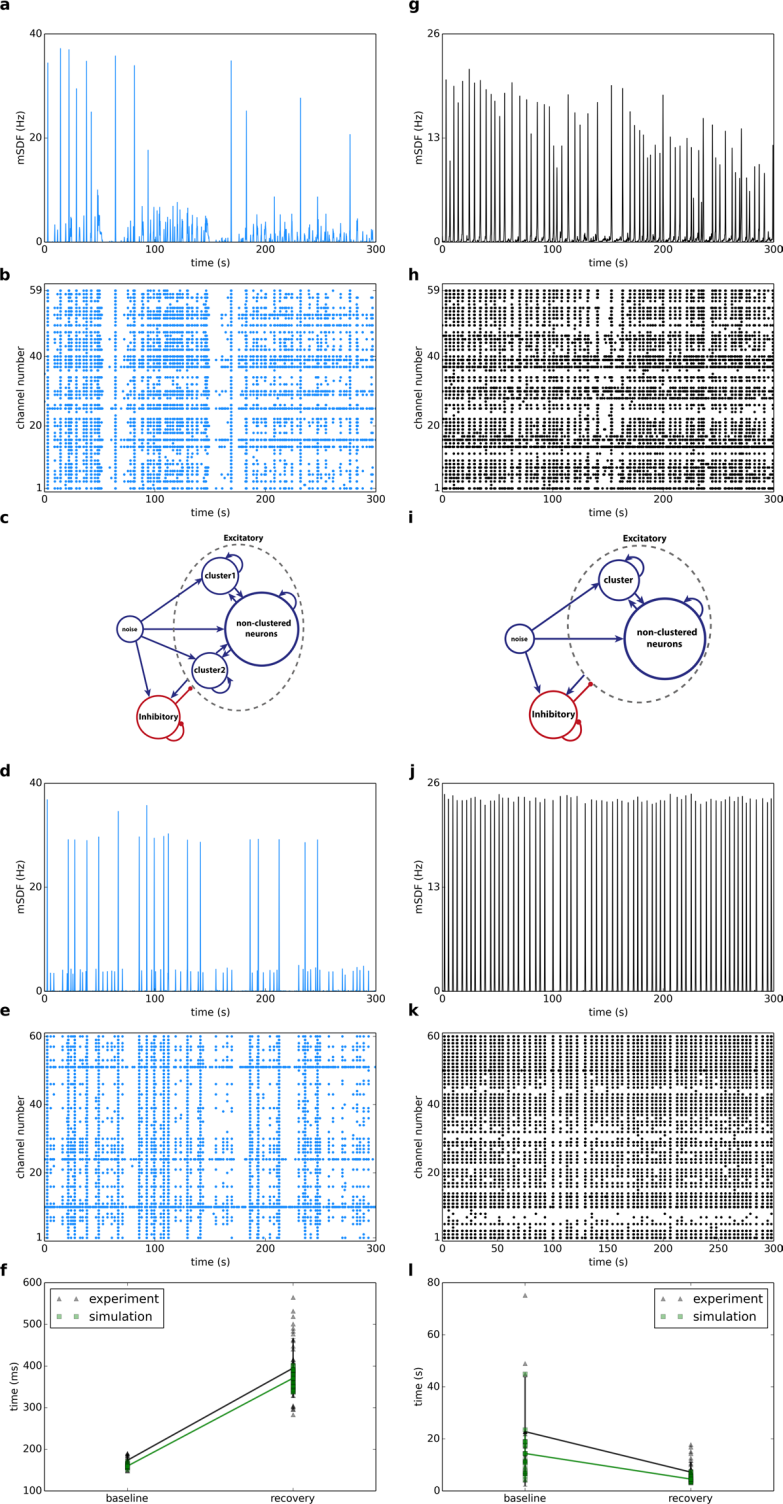
Computer Simulation of the network firing behavior of the second culture. Experimentally recorded mean spike density function and raster plot of the culture in culture in the baseline mode (**a**,**b**) is different with the recovery mode (**g**,**h**). In order to reproduce similar dynamics with the computer simulation we assumed that the network has two clusters in the baseline mode (**c**), while they merge into one cluster after the injection (**i**). Simulated mean spike density functions (baseline **d**, recovery **j**) and raster plots (baseline **e**, recovery **k**) are similar to the recorded data. Comparisons of burst durations (**f**) and interburst intervals (**l**) between experimental and simulation data indicate a very similar pattern.

We built a network with 900 inhibitory neurons and 3000 excitatory neurons (see Methods). To reproduce the dynamics of the recorded culture in Fig. 1, we embedded a cluster of 95 neurons in the excitatory population. Figure 6d shows the schematic of the network at baseline. Both synaptic weight and connection probability are higher inside clustered neurons compared to the connections between non-clustered neurons and between non-clustered and clustered neurons (see Methods). To display the results as multielectrode array recordings, we defined 60 channels. For each channel, we randomly picked 4 neurons from the network and aggregated their spikes. Figure 6a–c show the raster plots of channels and simulated mean firing rates (filtered with a Gaussian function, σ = 100 ms) at baseline. Simulated multiunit firing of a 9 second recording in Fig. 6a is shown at higher resolution in Fig. 6. We assume that increasing the firing rate of neurons after stimulation with the waking cocktail triggers long-term synaptic plasticity and modifies the synaptic weight between neurons. Therefore, this might lead to a new connectivity structure in the neuronal network. In this simulated culture, we suggest that the new structure has two clusters with 95 and 90 neurons (Fig. 6h). In other words, the stimulation adds another cluster in excitatory neurons population. Therefore, the culture exhibits a different oscillatory behavior (Fig. 6e,g). As observed in the experimental data, changes in burst duration (Fig. 6i) and inter-burst intervals (Fig. 6j) are very similar between the experimental and simulated data. Using a similar number of simulated inhibitory and excitatory neurons we also reproduced the behavior of another culture with a different connectivity structure (Fig. 7). During the baseline phase, we defined two clusters with 65 and 40 neurons. This structure showed a similar dynamics to the baseline recording (Fig. 7). To simulate the dynamics of the culture during recovery, we assumed that stimulation results in merging of the two clusters with a new and bigger cluster with 100 neurons (5 clustered neurons lost their connections to other neurons). This new structure reliably showed a network oscillation similar to the experimental data (Fig. 7). In summary, we showed that the network topology (number of neural clusters and size of each cluster) is a possible determinant of neuronal behavior and oscillations. We assumed that the different oscillatory behavior of each cluster is due to different topologies. Also, stimulation may change the topology of the network, which leads to different burst durations and inter-burst intervals at baseline and during recovery.

## Discussion

In this work we studied the network activity of cultured cortical neurons at baseline and during recovery after stimulation by a cocktail of waking neuromodulators. Our aim was to investigate if the behavior of the network during recovery (when slow oscillations reappear) shows homeostatic changes as seen during sleep in living animals. We found that during recovery the inter-burst interval is decreased while the burst duration is increased. Moreover, the power density in slow frequencies is increased together with the slope of UP and DOWN states. Our results clearly indicate that during recovery the neural network correlated activity shows a higher temporal and spatial synchrony, reminiscent of the patterns observed during recovery sleep after sleep deprivation *in vivo*. The overall changes during recovery are dose-dependent with stronger stimulations (1CCK) leading to larger differences, similar to longer wakefulness durations leading to larger sleep changes *in vivo*. We also show that neural trajectory method could trace temporal evolution of neuronal firing during recovery as a result of a higher synchrony with stronger phasic neural oscillation (UP and DOWN). Our simulations strongly suggest that the overall network behavior can be predicted by changes in activity of clusters within the network. One important finding resulting from *in vitro* preparations or isolated cortical islands is that neural networks default activity state is synchronized slow oscillations (sleep-like state)^18,20,22,23,25^. More importantly, continuous stimulation or inhibition cannot prevent the return to this default mode^25,31^. Note that in our experiments, the cultures were stimulated with a cocktail of neuromodulators without wash out. Although different neuromodulators might have different half-lives, signaling mechanisms, and time courses of feed-back induction^31^, our cultures are most probably nearly continuously stimulated. In an elegant experiment, cortical cultures were continuously stimulated with carbachol or noradrenaline leading to the disappearance of synchronous bursting that recovered within 24 hours^25^. Therefore, the default slow oscillation is regulated by homeostatic processes that play as strong attractors bringing the network activity back to its set-point. The underlying cellular and molecular mechanisms remain unknown. Obviously receptor desensitization might not be involved since for instance continuous cholinergic stimulation renders the cortical networks insensitive to noradrenergic stimulation^25^, although changes in overall receptor trafficking cannot be excluded. Recent observations also suggest that changes in firing rate are not compatible with transcriptional modifications^32^. Other mechanisms such as intracellular calcium or membrane homeostasis might be involved^22,33,35^. Interestingly, by using different methods and electrical or TNF alpha stimulation of cortical cultures, a similar pattern of homeostatic regulation was also observed^23^. The cellular mechanisms of recovery during sleep are poorly investigated. Nevertheless, detailed analysis of slow oscillations during human sleep at baseline and during recovery indicated very similar changes as reported here^15^. As opposed to the recent report^30^ by multiunit activity in freely behaving mice, where an increase in OFF state was reported (longer inactivity periods), both *in vivo* and our results indicate that recovery leads to more frequent UP and DOWN states^15^ with longer UP and shorter DOWN state. Also, experimental and computational data indicated that higher sleep need leads to steeper slope of the slow oscillation^16,17^ as reported here. In previous studies, the homeostatic recovery of firing rate was investigated mainly by inhibition, such as visual deprivation *in vivo*^42^ or GABA_B_-mediated silencing *in vitro*^43^, with *in vivo* findings favoring a cell-autonomous while *in vitro* results favoring a network homeostatic process. Nevertheless, even in the visual deprivation paradigm, the homeostatic regulation of firing rate set-point was found dramatically affected by ongoing network activity (wakefulness promoting and sleep inhibiting recovery)^42^. Obviously, neuronal activity depression requires activity (wakefulness) while over-activation requires sleep.

Whether the homeostatic mechanisms are cell autonomous or properties of networks remain controversial^42,43^. *In vitro* observations strongly favor the network hypothesis^43^. Also, the recent development of high density MEAs based on CMOS technology (with 4096 microelectroldes) revealed a high number of random firing activity while the overall network behavior was driven by synchronized bursts^44^. Excitatory cortical neurons are known to form privileged synaptic connections to form clusters^45^. We show that similar to intact cortex, the number of clusters, number of neurons in each cluster, synaptic weight, and connection probability inside and outside the clusters, as well as the amount of noise that each neuron receives affect the shape of the oscillations. Using our simulation model, we expected weak connections between neurons during the early days of cell cultures to be able to establish neural clusters and network oscillations. As the cell culture proceeds to maturation, connections are formed between neurons and stronger connections maintained by synaptic plasticity. These neurons form the clusters through long-term synaptic plasticity rules^46^ and the network produces oscillations during the baseline phase. Stimulation with our waking cocktail forces neurons to discharge at higher rates for a long period of time (nearly 24 h). Based on the firing pattern, synaptic plasticity modifies the structural connectivity between neurons leading to the formation of new clusters or the collapse of existing ones. The number of neurons in the cluster may also change. The new structure leads to a new oscillation (recovery mode) with different properties compared to the baseline oscillation (before stimulation).

A major discrepancy between sleep homeostasis as indexed by the EEG SWA and neuronal firing homeostasis is the large difference in their time course. While SWA shows a fast kinetics, typically within tens of minutes in rodents and a few hours in humans, network homeostasis, both *in vitro* and *in vivo* takes up to two days^42,43^.

Although the slow oscillation (<1 Hz) is at the basis of slow waves recorded by the EEG, other oscillations such as delta waves and spindles are critically modulated and/or generated by the cortico-thalamic network. How such oscillations are regulated remains unknown. Nevertheless, the spontaneous generation of such oscillations in more complex thalamo-cortical co-cultures (or interconnected through microfluidics devices) may be obtained and being subjected to detailed analysis as reported here.

## Methods

### Cell cultures

Cortical cultures were prepared from C57BL/6J mouse brains at embryonic days 18–20. The brain tissue was separated and dissected in a phosphate buffer solution containing HEPES, 33 mM glucose, and 40 mM sucrose. The isolated cortices were digested with a solution containing 50 U of papain for 30 min at 37 °C. Digestion was stopped by the addition of trypsin inhibitor for 10 min. Cells were then mechanically dissociated and plated in neurobasal medium supplemented with 2% B-27, 0.5 mM glutamax, and penicillin/streptomycin. Before seeding (200,000 neurons / MEA), the microelectrode array biosensors (MEAs; Multichannel Systems, Germany) were coated with 0.1% polyethyleneimine and 5 μg/ml of laminin to promote cell adhesion. Cultures were maintained in a humidified CO_2_ incubator (5% CO_2_, 37 °C) and half of the medium was changed once a week with the complete neurobasal medium. All cultures were recorded between 12 and 14DIV when stable burst-pause activity was observed^22^ and were either sham (H_2_O) stimulated or stimulated with a cocktail of neuromodulators: 1 μM NMDA, AMPA, kainate, ibotenic acid, serotonin, histamine, dopamine and noradrenaline; 10 μM carbachol; and 0.01 μM orexin^22^. This coctail (1CCK) was two-fold diluted to prepare the 0.5CCK. All experimental procedures were conducted in accordance with regulatory standards and approved by the Vaud Veterinary Office, Switzerland.

### Microelectrode Array (MEA) recording

Electrophysiological signals were acquired using the complete MEA60-BC system (Multichannel Systems, Germany). The set-up consists of a MEA 1060-Inv-BC amplifier integrating 60 channels and filter amplifiers with a bandwidth of 0.1 Hz–10 KHz and a gain of 1100. The set-up was connected to a computer equipped with a PCI data acquisition board and raw data were acquired and analyzed using MCRack software (Multichannel Systems, Germany). Primary neuronal cultures were seeded on standard MEA biosensors containing 59 planar TiN/SiN micro- electrodes (30 μm diameter, 200 μm interelectrode distances) plus one internal reference electrode (Fig. 1a). Spontaneous firing activity (Fig. 1b) was recorded after 2 weeks *in vitro* when a stable network activity was established (which appears approximately after 10 days in murine cortical cultures^22^). All recordings (300 seconds long) from MEAs were performed in a humidified CO_2_ incubator 10–15 minutes after the transfer of the MEAs into the recording stage. The raw signals were recorded at 25 kHz sampling frequency, high pass filtered at 200 Hz and low pass filtered at 2 kHz, and amplified spikes were isolated at 1 ms resolution. Several cultures were recorded at baseline and every 3 to 6 h after stimulation till dish-wide burst activity resumed at around 24 h (Fig. 2). Each recording lasted for 5 minutes. A total of 29 cultures were analyzed; 12 cultures were stimulated at high concentration (1CCK), 8 at low concentration (0.5CCK, half of the 1CCK concentration) and 9 were sham (H_2_O) stimulated.

### Spikes and synchronized bursts detection

Neuronal spikes were sorted from the biological noise using the threshold tool of MCRack software when the amplitude (peak-to-peak) of the extracellular potential exceeded a noise-based threshold set at 7 times the standard deviation of the noise for each MEA channel^47^. The spike time stamps were stored in the MCRack software. The recorded spike train is time-varying spontaneous multi-unit activity in the vicinity of each MEA electrode. The network activity is composed by both spikes and synchronized bursts (Fig. 1b). To calculate the network firing rate, we computed a spike density function (SDF) for each MEA electrode during the five minute long recordings. Briefly, spike trains were convolved by a Gaussian function with a total area of 1 and a width (SD) of 100 ms. The population firing rate or mean of SDF (mSDF) was then calculated by averaging the firing rate across all channels at each time point (Fig. 1e).

Primary cortical cultures are characterized by repetitive burst activity. Synchronized network activity across all channels were detected by using a method described in Mukai *et al*.^48^ and^49^. Briefly, the total number of spikes contained in a 100 ms time window were counted over all electrodes. By convolving the window on the spike train, a firing rate histogram was obtained over time. Finally, all the events exceeding a 40 spikes/window threshold were defined as a synchronized burst (Fig. 1f). In order to avoid any biased results due to inter-variability across cultures, all detection procedures for single spikes and synchronized bursts were tested in accordance to specific acceptance criteria as described in Novellino *et al*.^50^.

### Characteristics of synchronized burst activity

To characterize multiple features in the time domain, the detected burst-pause activity was analyzed during baseline and its variation during the recovery period (24 hours after stimulation). Different parameters were analyzed such as the number of spikes per channel, number of bursts, burst duration (BD: distance between raising and decaying segments of a burst at 25% of the maximum burst), Inter-Burst-Interval (IBI: distance between the maximum of 2 consecutive bursts), and the number of spikes per burst (Fig. 1f,j).

To detect burst’s slopes, we first normalized each burst activity distribution by dividing to the maximum of the burst amplitude. For each detected burst, the slope was defined as the first derivative of the normalized burst activity. The ascending and descending phases are denoted as initial and final slope, respectively.

It is believed that spiking activity is correlated across neurons in a population of neural networks. Cross-correlation is a method to detect the degree of interdependency (synchrony) between firing of paired neurons or electrodes^51–53^. For all pairs of MEA channels, the cross-correlation was computed as follows. First, all spikes were placed into bins of 1 ms. Then, within a time window (T = 1000 ms), ${C}_{ij}(\tau )=(\,\sum _{\tau =0}^{T}{x}_{i}[s]{x}_{j}[s+\tau ])$ was calculated where s is the starting time of the window and *x*_*i*_[*s*] is the number of spikes filtered by a Gaussian Kernel with a width (SD) of 100 ms in time interval [s, s + 1] ms in channel i. This value (*C*_*ij*_(*τ*)) was divided to $\sqrt{{C}_{ii}(0)\,{C}_{jj}(0)}$, and the maximum value was selected: *C*_*ij*_(*τ*) over *τ* = 1, 2, 3, ..., *T* for all time windows. The resulting cross-correlation matrix was averaged per culture and cross-correlations were compared between conditions (baseline/recover) and treatments (CCK1, CCK0.5, and sham) by a two-way repeated measures ANOVA. Hierarchical clustering analysis was performed using the linkage method and the Scipy software package^54^.

### Spectral Analysis

The spectral components are lost during the extraction of temporal features of the signal. To better understand the network oscillation, a fast Fourier transform (FFT) was performed on burst-paused mSDF population firing rate with a 100 ms bin width at 1 kHz sampling rate^55^. Before FFT, the DC component was removed by subtracting the mean from the data. The obtained power spectrum across all cultures at baseline showed a typical dominant frequency with the highest peak within the slow oscillation band frequency (≤1 Hz).

### Neural Trajectory

To consider the network properties at the population level, there are two concerns. First, salient features of the channel responses may be masked by averaging across channels. Second, it is difficult to characterize multiple spatiotemporal features of a high-dimensional oscillating network. To address these concerns, we assessed representative 2-d linear projections of the 60 channel responses. For example, principal component analysis (PCA) can be applied to the channel responses to assess the top two principal components. While this projection captures the greatest amount of variance, it is not guaranteed to capture the oscillatory nature of the data. Instead, we used the DataHigh software^34^ to view many 2-d projections of the data. DataHigh first smooths the spike counts of each channel across time with a 50 ms Gaussian kernel. Next, DataHigh applies PCA to the smoothed channel responses (60-dimentional spike count vectors) and takes the top K dimensions that explain 90% of variance (where K is typically greater than 2). This helps to remove dimensions with small amount of variance. DataHigh then allows the user to view many 2-d projections of the K principal components by plotting the data as a ‘neural trajectory’, where two consecutive time points are connected by a line. The neural trajectory emerges as a circular oscillation which corresponds to the beginning and the end of an entire trial (a five minute channel firing rate which starts and stops at the same bistable neural state). Thus, the neural trajectory represents the temporal evolution of the channel responses, which may oscillate from one region of firing rate space to another. These regions correspond to states, where an ‘UP’ state corresponds to elevated firing rates, whereas ‘DOWN’ state corresponds to low firing rates.

### Simulation

For the neuron model, we used a current-based generalized integrate-and-fire (GIF) model^56^, which implements spike-frequency adaptation. It is shown that the GIF model is able to capture both subthreshold dynamics of membrane potentials and spikes recorded from neurons in the cortex with high accuracy during current injection^56^. The model describes the dynamics of the membrane potential *V*(*t*) by the differential equation:

1 $$C\frac{dV(t)}{dt}=-\,{g}_{{\rm{L}}}(V(t)-{E}_{{\rm{L}}})-\sum _{\widehat{{t}_{j}} < t}\eta (t-\widehat{{t}_{j}})+I(t)$$

where *I*(*t*) is the input current. *C*, *g*_L_ and *E*_L_ are parameters of the neuron model and $\{\widehat{{t}_{j}}\}$ are the spike times. In case of spike emission, a current with the shape *η*(*t*) is triggered. The neuron goes through a refractory period with the duration of *τ*_ref_ and the membrane potential is reset to *V*_reset_. Spikes are produced stochastically with the firing intensity

2 $$\lambda (t)={\lambda }_{0}\,\exp (\frac{V(t)-{V}_{{\rm{T}}}(t)}{{\rm{\Delta }}V})$$

where ${\lambda }_{0}$ and $\Delta V$ are the parameters of the firing intensity. ${V}_{{\rm{T}}}(t)$ is firing threshold:

3 $${V}_{{\rm{T}}}(t)={{V}_{{\rm{T}}}}^{\ast }+\sum _{\widehat{{t}_{j}} < t}\gamma (t-\widehat{{t}_{j}})$$

where ${{V}_{{\rm{T}}}}^{\ast }$ is a constant. After each spike emission, a shape *γ*(*t*) is added to the firing threshold. Supplementary Table 1 summarizes the parameters and shapes of *η*(*t*) and *γ*(*t*) used for excitatory and inhibitory neurons in the simulations. Neuron parameters are extracted from experiments performed in the mouse barrel cortex^56^.

Neurons only receive synaptic current as the input (*I*(*t*)). The input received by neuron *i* is generated by the spikes of synaptically connected neurons:

4 $${I}_{i}(t)=\sum _{j}{w}_{ij}\,\sum _{f}\alpha (t-{\hat{t}}_{f,j})=\sum _{j}{w}_{ij}{\int }_{0}^{\infty }\alpha (t){S}_{j}(t-s)ds$$

where *w*_*ij*_ is the synaptic weight of connection from neuron *i* to *j*. ${\hat{t}}_{f,j}$ is the *f*^*th*^ spike of neuron *j*. Postsynaptic current shape is described by:

5 $$\alpha (t)=w{e}^{-(t-{\rm{\Delta }})/{\tau }_{{\rm{syn}}}}$$

for $t\ge {\rm{\Delta }}$ where ${\tau }_{{\rm{syn}}}$ is synaptic time constant. The transmission delay (Δ) for all synapses is 2 ms. ${S}_{j}=\sum _{f}\delta (t-{\hat{t}}_{f})$ is the spike train of neuron *j* where *δ* denotes the Dirac *δ*-function. The synaptic weights, connection probabilities and time constants between different subgroups of neurons are different. Supplementary Table 2 shows the parameters used for building the network. In a previous work^57^, we investigated the role of these different network elements in oscillation properties.

We assume that each neuron receives noise, beside the synaptic input from other neurons. This noise is modeled with a stochastic Poisson input. Supplementary Table 3 displays the properties of the Poisson input each neuron receives. All simulations were performed by Brian simulator^58^.

### Statistical analysis

All analyzed parameters were first tested for normality by the Kolmogorov-Smirnov test. The extracted network-burst parameters were first expressed relative to the sham condition followed by Student’s paired t-test. The power spectra were compared among conditions by 2-way ANOVA followed by Tukey test. The cross-correlation coefficients were compared by 2-way repeated measures ANOVA followed by Tukey test. Statistical significance level was set at p < 0.05.

### Availability of data and material

All data generated or analyzed in the study are included in this published article.

## Electronic supplementary material

Supplementary Dataset 1
