# Supplementary material for: In vitro Cortical Network Firing is Homeostatically Regulated: A Model for Sleep Regulation
Source: Sci Rep. 2018 Apr 19;8:6297. doi: 10.1038/s41598-018-24339-6 (PMC5908861; doi:10.1038/s41598-018-24339-6)
Supplement: Supplementary file 1 — Supplementary Dataset 1 [file 41598_2018_24339_MOESM1_ESM.pdf]

IN VITRO CORTICAL NETWORK FIRING IS HOMEOSTATICALLY REGULATED: A MODEL FOR SLEEP REGULATION

Sohrab Saberi-Moghadam[1], Alessandro Simi[1]*, Hesam Setareh[2]*, Cyril Mikhail[1]

and Mehdi Tafti[1,3#]

Supplementary Table 1 Parameter of GIF neuron model used in all simulations.

Parameter	Excitatory neurons	Inhibitory neurons
C (pF)	83.1	46.1
g_{L} (nS)	3.7	6.6
E_{L} (mV)	-67.0	-71.2
$\eta(t)$ (pA)	$11.7e^{-t/53.8ms} + 1.8e^{-t/640.0ms}$	$31.8e^{-t/11.5ms} + 1.6e^{-t/500.1ms}$
$\gamma(t)$ (mV)	$56.7e^{-t/57.8ms} - 6.9e^{-t/218.2ms}$	$5.6e^{-t/57.8ms} + 0.6e^{-t/473.7ms}$
λ_0 (s^{-1})	0.1	0.1
ΔV (mV)	1.4	0.6
V_{T}^* (mV)	-55.8	-48.1
τ_{ref} (ms)	4.0	4.0
V_{reset} (mV)	-36.7	-48.4

Supplementary Table 2 Network parameters used for each simulation. Excitatory neurons include cluster and non-clustered neurons. Connections between inhibitory neurons and cluster neurons are same as connections between inhibitory neurons and non-clustered neurons.

Simulation	Connection	Connection Probability	τ_{syn} (ms)	w (pA)
First culture baseline mode (Fig. 6)	cluster ➔ cluster	50%	16.3	18.6
	cluster ➔ non-clustered neurons	15%	16.3	4.4
	non-clustered neurons ➔ cluster	10%	16.3	4.4
	non-clustered neurons ➔ non-clustered neurons	15%	16.3	4.4
	excitatory ➔ inhibitory	37%	6.9	14.8
	inhibitory ➔ excitatory	25%	1.3	39.6
	inhibitory ➔ inhibitory	35%	6.9	10.1
First culture recovery mode	cluster1 ➔ cluster1	50%	16.3	18.6
	cluster2 ➔ cluster2	50%	16.3	18.6
	cluster1 ➔ cluster2	10.5%	16.3	2.6
	cluster2 ➔ cluster1	10.5%	16.3	4.4
	cluster1 ➔ non-clustered neurons	15%	16.3	4.4
	cluster2 ➔ non-clustered neurons	15%	16.3	3.5

	non-clustered neurons ➔ cluster1	5%	16.3	4.4
	non-clustered neurons ➔ cluster2	15%	16.3	4.4
	non-clustered neurons ➔ non-clustered neurons	15%	16.3	4.4
	excitatory ➔ inhibitory	37%	6.9	14.8
	inhibitory ➔ excitatory	25%	1.3	39.6
	inhibitory ➔ inhibitory	35%	6.9	10.1

Simulation	Connection	Connection Probability	τ_{syn} (ms)	w (pA)
Second culture baseline mode (Fig. 7)	cluster1 ➔ cluster1	55%	16.3	18.6
	cluster2 ➔ cluster2	55%	16.3	18.6
	cluster1 ➔ cluster2	5%	16.3	3.5
	cluster2 ➔ cluster1	5%	16.3	3.5
	cluster1 ➔ non-clustered neurons	18%	16.3	3.1
	cluster2 ➔ non-clustered neurons	18%	16.3	2.6
	non-clustered neurons ➔ cluster1	18%	16.3	4.4
	non-clustered neurons ➔ cluster2	18%	16.3	4

	non-clustered neurons ➔ non-clustered neurons	18%	16.3	4.4
	excitatory ➔ inhibitory	18.6%	6.9	14.8
	inhibitory ➔ excitatory	25%	1.3	39.6
	inhibitory ➔ inhibitory	35%	6.9	10.1
Second culture recovery mode	cluster ➔ cluster	45%	16.3	18.6
	cluster ➔ non-clustered neurons	12%	16.3	2.4
	non-clustered neurons ➔ cluster	12%	16.3	3
	non-clustered neurons ➔ non-clustered neurons	18%	16.3	4.4
	excitatory ➔ inhibitory	18.6%	6.9	14.8
	inhibitory ➔ excitatory	25%	1.3	39.6
	inhibitory ➔ inhibitory	35%	6.9	10.1

Supplementary Table 3 Parameters of external Poisson noise. Each neuron receives independent Poisson input with constant rate r and a synaptic weight w.

Simulation	Connection	r (Hz)	τ_{syn} (ms)	w (pA)
First culture baseline mode	Poisson ➔ cluster	95	16.3	16
	Poisson ➔ non-clustered neurons	95	16.3	9
	Poisson ➔ inhibitory	100	6.9	30
First culture recovery mode	Poisson ➔ cluster1	95	16.3	16
	Poisson ➔ cluster2	100	16.3	12
	Poisson ➔ non-clustered neurons	95	16.3	9
	Poisson ➔ inhibitory	100	6.9	30
Second culture baseline mode	Poisson ➔ cluster1	48	16.3	28
	Poisson ➔ cluster2	95	16.3	19
	Poisson ➔ non-clustered neurons	95	16.3	4
	Poisson ➔ inhibitory	100	6.9	30
Second culture recovery mode	Poisson ➔ cluster	66	16.3	23
	Poisson ➔ non-clustered neurons	95	16.3	4
	Poisson ➔ inhibitory	100	6.9	30
